# Elemental compositions of particulate matter retained on air condition unit’s filters at Greater Doha, Qatar

**DOI:** 10.1007/s10653-019-00304-8

**Published:** 2019-05-03

**Authors:** Mohamed M. Mahfouz, Oguz Yigiterhan, A. E. Elnaiem, Hassan M. Hassan, Balint Alfoldy

**Affiliations:** Environmental Science Center (ESC), Qatar University, H10-Zone 3-B113, P.O. Box 2713, Doha, Qatar

**Keywords:** Indoor air quality, Urban air pollution, Aeolian dust, Households dust, AC filter dust, Trace elements, Enrichment factors

## Abstract

Elemental composition of airborne dust samples retained by internal filters of air condition units (ACUs) was determined at 12 locations of Doha city, state of Qatar. Twenty-four elements: Al, Ca, Mg, Fe, Na, K, Ti, Zn, P, Sr, Mn, Ba, Cu, Cr, Ni, Pb, V, Mo, Li, Co, Sb, As, Cd, Be, were analysed by ICP-OES technique after acid digestion of the samples. The analysed components reflect 20.6% of the total sample mass. Similar or lower concentration values have been found for As, Cd, Cr, Cu, Mn, Ni, Pb, V, Zn, Al, and Fe compared to the international context of upper crust concentrations, NIST SRM (urban dust), published local dust information of outdoor, and surface terrestrial deposit (STD) counted for 7.2, 0.7, 91.8, 192.8, 369.7, 68.6, 65.3, 52.1, 824.3, 19,791, 20,508 mg/kg, respectively. The coefficient of correlation (*p* ≤ 0.05) showed significant association of ACUs dust elemental compositions with the main components of the local earth crust and surface deposits, ranging from the lowest 0.77 (Mg–Fe) to the highest 0.98 (Al–Fe), while Ni and V, typical anthropogenic pollutants, are also strongly correlated (0.86). These strong correlation relationships can be interpreted as the contribution of outdoor particulate to the indoor dust. Dendrogram of metal/Al ratios, based on Euclidean distance calculation and average linkage clustering method, distinguished three typical groups. Studying the enrichment factors of the three groups indicated elevated levels of Zn (131), Pb (49), Cu (32), Cd (8) and Ni (5) found indoors compared to the background composition of STD especially at locations in the industrial zone. The major elemental composition of the samples reflects the typical mineral composition of the local dust, while the trace composition demonstrates the influence of indoor sources. The collected ACU filter dust samples show significant contribution of outdoor mineral particles, non-exhaust traffic emission, industrial sources, as well as the influence of indoor activity such as smoking.

## Introduction

The focus on heavy metal pollution in household dust has increased recently, as metallic elements can have a significant effect on human health and the ecosystem. Heavy metals can be toxic, persistent, bioaccumulative, and have the ability for biomagnification (Shi et al. 2011). These elements adsorbed by dust particles can easily be inhaled, ingested or absorbed via dermal contact (Glorennec et al. 2012; Sah et al. 2018; Sharma et al. 2018). Their accumulation within the tissue and internal organs (Zheng et al. 2010) can affect the central nervous system and may lead to the promotion of other diseases (Faiz et al. 2009). For instance, Li et al. (2001) demonstrated that low level lead (Pb) exposure can be detrimental to enzymic systems, as well as brain and blood production within the human body, whereas long-term exposure may affect mental development in children (Li et al. 2001; Sezgin et al. 2004) .

In hot, arid environments people tend to spend most of the time indoors (either at the workplace or at home) (Habil et al. 2016). Thus, the quality of the indoor air can have a direct impact on their health. The quality of air in Qatar, particularly in its major urban centres, has been declining for the last decades due to country’s ever-increasing industries, constructions, and vehicle numbers (Ferwati et al. 2017; Saraga et al. 2017). Elevated indoor heavy metals concentrations can be attributed to outdoor sources such as heavily traffic roads (Cao et al. 2018; Li et al. 2001) and proximity to industrial sites (Parveen et al. 2018). Moreover, natural sources of air pollution are also crucial in the country based on its desert climate, where dust storms can significantly affect air quality. A recent report released in 2013 by the Qatar Ministry of Development, Planning and Statistics (MDPS) highlighted that the pollution levels in Qatar frequently exceed the WHO recommendations as well as Qatar’s air quality targets (MDPS 2014).

Air condition units (ACUs) are used extensively in residential and commercial buildings almost all the year round in Qatar. The analysis of the dust, accumulated on their filters, can provide valuable information about the indoor air quality. Thus, researching the heavy metal content of indoor dust becomes essential and will help to understand possible changes of urban environmental quality, caused by intensive anthropogenic activities (Han and Lu 2017) in the Gulf region.

In this research, we aim to evaluate the metal content of retained dust particles and their influence on indoor air quality in Doha. Dust samples were collected from ACUs filters, and the elemental composition of the particulate matter (PM) was determined by ICP-OES analysis. Dust samples were collected at different key locations within the city, representing traffic influenced and background locations as well as industrial sites.

## Materials and methods

### Sample collection

**Fig. 1:**
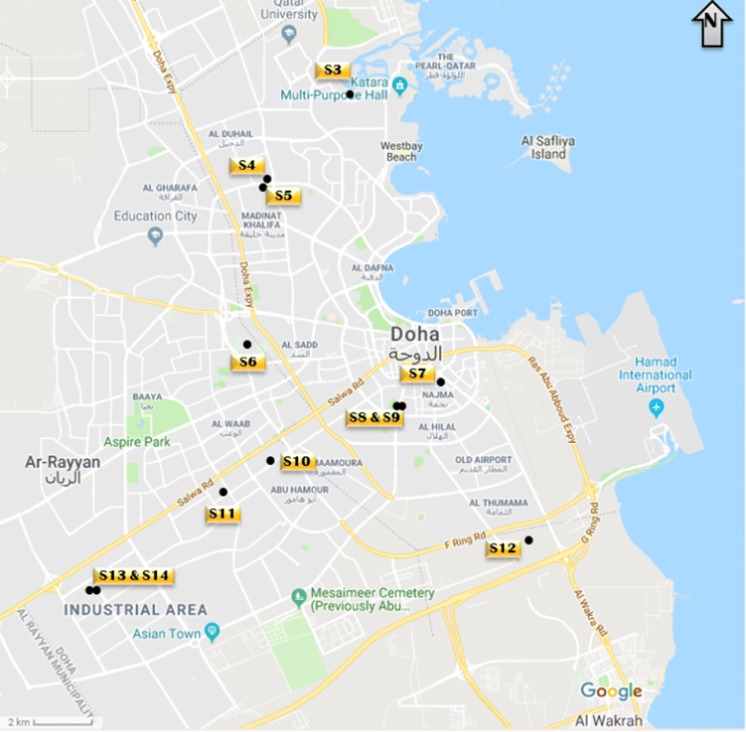
Sampling locations within the Greater Doha area (courtesy of Google Earth 2018)

Indoor house dust samples (*n* = 12) accumulated on air condition filters were collected from residential and commercial buildings, representing different environmental conditions, within Doha (Fig. 1). Each sampling location was carefully selected to reflect differences in the influences of local traffic, construction and industrial activity, or rural residency along the eastern coast of Doha, where most of the country’s population reside. Geographical coordinates and description of the sampling sites are given in Table 1.

**Table 1:** Locations and description of sampling sites

Sample #	Location (latitude, longitude)	Site description/traffic status	Remark
S3	25°21′47.03″N 51°29′49.04″E	Residential, light traffic	Nonsmoking
S4	25°20′20.87″N 51°27′47.60″E	Residential, light traffic	Nonsmoking
S5	25°20′16.58″N 51°27′48.35″E	Residential, light traffic	Smoking
S6	25°17′35.55″N 51°25′52.27″E	Residential, heavy traffic	Nonsmoking
S7	25°16′37.30″N 51°32′14.70″E	Residential, heavy traffic	Nonsmoking
S8	25°16′27.52″N 51°31′44.22″E	Residential, heavy traffic	Nonsmoking
S9	25°16′27.51″N 51°31′43.70″E	Residential, heavy traffic	Nonsmoking
S10	25°15′13.92″N 51°29′21.17″E	School office, heavy traffic	Nonsmoking
S11	25°14′16.95″N 51°27′27.67″E	School office, light traffic	Nonsmoking
S12	25°14′10.46″N 51°33′35.28″E	Residential, heavy traffic	Nonsmoking
S13	25°12′16.04″N 51°25′06.14″E	Industrial office, heavy traffic	Smoking
S14	25°12′16.33″N 51°25′05.89″E	Industrial office, heavy traffic	Smoking

The sampling period was predefined to obtain maximum loadings of the PM during the dry summer season of 2015. The ACUs filter samples were exposed for 2–3 months before sampling. Operation of the ACUs creates a low-pressure area, similar to a vacuum, attracting particles from the entire room and forming a homogenised dust that can represent the actual contamination level (Tan et al. 2007). The deposited particles were extracted from the filter tissues by gently shaking, and the falling out particles were collected on a polycarbonate sheet. The dust samples were transferred into acid-rinsed glass jars and kept at low temperature (− 4.0 °C) before digestion and analysis.

### Sample preparation

Collected samples were dried in an air oven set to 105 °C for 12 h. The dried samples were then homogenised and stored in an airtight desiccator till digestion.

0.25 g of each sample was carefully weighed into a Teflon tube, and 9 ml of SuporaPure^®^ nitric acid (6 M) was added to each tube. The tubes were placed into a HotBloc^®^ digestion block set to 95 °C; the set temperature was maintained for 30 min after which 3 ml of SupraPure^®^ hydrofluoric acid (16 M) was added to each vessel. After a further 30 min at 95 °C, the temperature was ramped to 135 °C and held for 60 min after which another temperature increase was applied (150 °C) until the sample dehydrated.

A 3 ml aliquot of nitric acid was added to each dry sample and diluted with 40 ml of double deionised water (DDW). The solution was allowed to boil until it was clear. The sample was then allowed to cool and further diluted to 100 ml using DDW. The samples were then filtered through a 0.22 µM syringe filter before injection and analysis via ICP/OES.

### Quality assurance

A certified marine sediment reference material (CRM) PACS3 for trace metals and other constituents, originated from National Research Council Canada (NRCC), was used for the elemental analysis. Quality Control was conducted using reagent blanks, duplicate samples, spiked samples, and PACS-3 CRM at every ten samples. These CRMs were verified within 5% of their expected values for all elements. Table 2 shows the recovery detected (96–99%) for some selected heavy metals.

**Table 2:** Recoveries of selected analytes in PACS3 reference sediment

	Analysis 1 (mg/kg)	Analysis 2 (mg/kg)	Avg. value (mg/kg)	Certified value (mg/kg)	Recovery (%)
*Heavy metals (CRM used PACS3)*					
As	29.5	28.9	29.2	30.3	96.4
Cd	2.11	2.18	2.15	2.23	96.2
Cr	90.8	90.0	90.4	91.6	98.7
Cu	311.8	319.1	315.5	327.0	96.5
Ni	40.1	37.7	38.9	39.9	97.5
Pb	179.2	189.2	184.2	188.0	98.0

In almost all cases, the average measured values were within the 95% confidence limits of the certified values, and thus the accuracy determined from this approach was comparable to or better than the precision. Procedural blanks were prepared using the same analytical procedure and reagents, but without adding a sample in the digestion vials. All samples analyses were carried out triplicate and analysed in ESC ISO 17,025 accredited laboratories. The limit of detection was defined as three times of the standard deviation of the blank readings.

### Environmental indexing

The enrichment factor (EF) is one of the most common environmental indices applied to distinguish the anthropogenic and natural sources of the elements in the sample based on their ratio to the primary component in the background soil composition. It is used on a broad field of environmental studies such as sediment and water sciences, and air quality research as an indicator of pollution (Gomes and Gillette 1993; Sutherland 2000; Adamo et al. 2005; Abrahim and Parker 2008; Rushdi et al. 2013; Shelley et al. 2014).

In this work, we calculated the EF for metals (Me) using aluminium as a reference due to its terrestrial origin and dominance in the earth’s crust composition within Qatar (Yigiterhan et al. 2018).

1 $${\text{E}}{{\text{F}}_{{\text{Me}}}} = {\left( {\frac{{{\text{Me}}}}{{{\text{Al}}}}} \right)_{{\text{ACU}}\;{\text{dust}}}}/{\left( {\frac{{{\text{Me}}}}{{{\text{Al}}}}} \right)_{{\text{bcgk}}}},$$

where $\left( {\frac{{{\text{Me}}}}{{{\text{Al}}}}} \right)_{{{\text{ACU}}\,{\text{dust}}}}$ is the concentration ratio of Me and Al in the AC dust sample, and $\left( {\frac{{{\text{Me}}}}{{{\text{Al}}}}} \right)_{\text{bcgk}}$ is their concentration ratio in the background soil.

EF value higher than 10 (EF >10) indicates the anthropogenic origin of the element, while EF<10 is attributed to natural composition (Biegalski et al. 1998; Nazir et al. 2011). The detailed classification of EF was given in Shaari et al. (2015).

## Results and discussion

### Elemental composition of the indoor dust

The chemical analysis of ACUs filter dust samples was conducted for 24 elements by ICP-OES technique; the detailed results of the analysis are summarised in “Appendix 1” section. The average elemental compositions of the samples are presented in Table 3. The compositions of indoor dust were compared to upper continental crustal concentrations (UCC) given by Rudnick and Gao (2003), and other local dust samples such as outdoor dust (OD), road dust (Rd) and terrestrial surface deposit (STD) collected at urban and background locations of Qatar (Yigiterhan et al. 2018) in order to determine the enrichment relative to the background composition. For a wider reference, mass concentrations of the same concerned elements in the 1648a standard reference material (SRM, urban dust) by the National Institute of Standards and Technology (NIST) are also included. The last row of the table shows the average total mass percentages of the analysed elements.

**Table 3:** Mean concentrations (mg/kg) of 24 elements detected in ACUs filters samples compared to published data of upper continental crust (UCC), outdoor dust (OD), road dust (Rd), surface terrestrial deposits (STD), and urban dust (1648a) SRM from NIST

Element	ACUs dust	UCC^a^	OD^b^	Rd^b^	STD^b^	1648a^c^
Al	19,812	81,500	23,700	22,200	25,600	34,300
Fe	20,504	39,176	14,100	6700	11,200	39,200
Ca	117,669	25,657	137,000	139,000	153,000	50,840
Mg	24,179	14,995	31,600	32,800	30,800	8130
Na	11,242	24,259	20,900	13,000	17,200	4240
K	7631	23,243	6200	6700	7600	10,056
Ti	1728	3836	–	–	–	4021
Zn	824.3	71.0	184.0	357.0	25.3	4800
P	624.6	327.3	415.0	349.0	209.0	–
Sr	427.8	350.0	732.0	653.0	741.0	215
Mn	369.8	774.5	279.0	272.0	319.0	790
Ba	314.9	550.0	209.0	292.0	233.0	–
Cu	192.9	25.0	38.4	162.0	13.0	610
Cr	91.8	35.0	62.3	63.9	82.0	402
Ni	68.7	20.0	51.4	40.0	28.3	81.1
Pb	65.3	20.0	10.9	9.7	5.3	6550
V	52.1	60.0	43.6	43.3	37.2	127
Mo	15.1	1.5	5.5	7.1	1.6	–
Li	12.6	20.0	19.8	9.7	11.8	–
Co	12.3	10.0	6.0	7.7	5.4	17.9
Sb	12.9	0.4	–	–	–	45.4
As	7.2	1.5	2.6	–	2.8	115
Cd	0.7	0.1	0.1	–	0.1	73.7
Be	0.5	3.0	0.4	0.4	0.3	–
Σ (%)	20.6	–	23.6	23.3	24.6	16.5

The total mass percentages of the measured elements are also given

^a^Rudnick and Gao (2003), ^b^Yigiterhan et al. (2018), ^c^NIST SRM

It is noted from the table that the analysed elements refer about 23.3–24.6% of the total mass of OD, Rd and STD samples, while 20.6% of the ACUs samples. The missing mass of the samples can be attributed to the unidentified major components of the minerals such as carbon and oxygen, as well as silica that could not be measured due to the analytical circumstances. Also, the organic components of the samples (Mahfouz et al. 2018) could have a significant contribution in the total sample mass. Supposing that the mineral composition of the ACUs filter is similar to the outdoor samples, the lower total mass percentage could refer to higher organic content due to especially indoor sources. The analytical results demonstrate the particular nature of the local airborne dust compared to typical urban air pollution, which reflected by the urban dust SRM. The local dust contains Ca and Mg at higher, while Al and Fe at lower concentrations than the SRM. Also, the traffic emission-related elements (Cu, Zn, Pb) are more enriched in the SRM. The total mass of the analysed elements is 16.5% in the SRM due to its different mineralogical composition (more silicate less calcite) and the higher percentage of organic compounds.

Eleven common elements were found in different publications about ACUs filter samples from the wider region of Asia, which are compared with Qatari indoor dust, presented in Table 4 (Abbasi and Tufail 2013; Al-hemoud et al. 2017; Huang et al. 2014; Siddique 2011). Our findings were within the range of the published values, except Al (1.9%), Fe (2.0%), V (52.1 ppm) and As (7.2 ppm) which were lower than literature average. In this study, average Zn concentration was found at 824 ppm, which is almost equal to the highest literature value given by Tufail et al. (2013). High Zn concentration can be attributed to outdoor anthropogenic activities such as industrial emissions and non-exhaust traffic emissions like tire debris, brake pads and road surface wear (Grigoratos and Martini 2015), but also indoor sources (Tunno et al. 2016). Similar to Zn, significant enrichment of Cu, Mo and Ni is more associated with traffic-related dust rather than local origin or transported dust (Thorpe and Harrison 2008).

**Table 4:** Mean concentration of selected trace (mg/kg) and major elements (vol%) in the ACUs filter from Qatar and other countries

Location	References	Concentrations (mg/kg)										
		As	Cd	Cr	Cu	Mn	Ni	Pb	V	Zn	Al	Fe
Qatar (this study)	Mean	7.2	0.7	91.8	192.9	369.8	68.7	65.3	52.1	824.3	19,812	20,504
Kuwait	Al-hemoud et al. (2017)	8.0	–	78.7	–	–	–	38.7	78.2	–	33,395	32,796
Pakistan	Tufail (2011)	7.7	–	77.5	–	579	–	–	–	452	51,187	33,307
Pakistan	Abbasi and Tufail (2013)	42.8	8.4	93.0	156.9	–	47.8	145.8	–	890.0	–	–
China	Huang et al. (2014)	17.1	0.4	188	68.4	321.2	94.9	699.1	–	344.2	–	–

(−) Not available

In order to compare the elemental distributions between different locations, Pearson correlation coefficients were calculated at 10 locations. The two samples from the industrial zone (S13 and S14) were kept out from the correlation analysis due to their outlying high concentration values that would bias the correlation. The correlation coefficients are presented in Table 5 at a significance level of *p* ≤ 0.05 and showed strong correlation between 8 elements (Al, Mg, Fe, Mn, Ni, V, Li and Be) with correlation coefficients ranging from the lowest 0.77 (Mg–Fe) to the highest 0.98 (Al–Fe). Moreover, Mg and V are strongly correlated with Ba (0.84 and 0.81), V–Ni (0.86), while Mn and Li with As (0.81 and 0.71). Al, Mg, Fe, Mn are the main components of the local earth crust and surface deposits (Yigiterhan et al. 2018), while Ni and V are typical anthropogenic pollutants originated from oil combustion (Visschedijk et al. 2012). The strong correlation of these elements can be interpreted as the contribution of outdoor dust for the indoor dust samples.

**Table 5:** Correlation coefficients between the components measured in 10 ACUs filter dust samples with 95% level of confidence

	Al	Ca	Mg	Fe	Na	K	Ti	Zn	P	Sr	Mn	Ba
Al	1											
Ca	0.23	1.00										
Mg	0.79	0.52	1.00									
Fe	0.98	0.33	0.77	1.00								
Na	− 0.38	0.29	− 0.07	− 0.39	1.00							
K	0.60	0.22	0.37	0.58	0.26	1.00						
Ti	0.22	0.52	0.25	0.33	− 0.06	0.03	1.00					
Zn	− 0.31	0.28	− 0.14	− 0.24	0.03	− 0.38	0.66	1.00				
P	0.57	0.10	0.47	0.54	− 0.18	0.39	0.30	0.31	1.00			
Sr	0.32	0.95	0.67	0.37	0.39	0.28	0.36	0.15	0.16	1.00		
Mn	0.94	0.38	0.85	0.94	− 0.14	0.64	0.23	− 0.34	0.45	0.49	1.00	
Ba	0.62	0.74	0.84	0.66	− 0.04	0.22	0.55	0.03	0.30	0.77	0.68	1.00
Cu	− 0.46	− 0.03	− 0.20	− 0.40	− 0.23	− 0.88	0.15	0.54	− 0.11	− 0.10	− 0.44	− 0.08
Cr	0.43	0.83	0.46	0.51	0.07	0.36	0.71	0.45	0.48	0.74	0.43	0.68
Ni	0.90	− 0.01	0.63	0.89	− 0.48	0.36	0.23	− 0.36	0.37	0.06	0.84	0.51
Pb	− 0.24	0.33	− 0.14	− 0.11	− 0.34	− 0.56	0.64	0.69	0.05	0.11	− 0.29	0.25
V	0.94	0.39	0.89	0.94	− 0.25	0.52	0.38	− 0.23	0.51	0.47	0.94	0.81
Mo	0.01	− 0.12	0.23	− 0.04	0.01	− 0.12	− 0.10	0.37	0.46	0.00	0.06	− 0.19
Li	0.97	0.23	0.81	0.95	− 0.27	0.63	0.17	− 0.33	0.55	0.34	0.98	0.57
Co	0.54	0.47	0.45	0.63	− 0.11	0.31	0.92	0.41	0.43	0.38	0.54	0.63
Sb	− 0.41	0.41	− 0.36	− 0.29	0.09	− 0.35	0.54	0.41	− 0.49	0.19	− 0.39	0.15
As	0.63	0.45	0.58	0.66	0.29	0.66	0.02	− 0.48	0.12	0.58	0.81	0.50
Cd	− 0.29	− 0.12	− 0.40	− 0.24	0.19	0.30	0.11	0.06	0.06	− 0.26	− 0.28	− 0.25
Be	0.96	0.12	0.64	0.95	− 0.37	0.70	0.15	− 0.31	0.60	0.19	0.90	0.42

	Cu	Cr	Ni	Pb	V	Mo	Li	Co	Sb	As	Cd	Be
Al												
Ca												
Mg												
Fe												
Na												
K												
Ti												
Zn												
P												
Sr												
Mn												
Ba												
Cu	1.00											
Cr	− 0.16	1.00										
Ni	− 0.27	0.16	1.00									
Pb	0.71	0.38	− 0.16	1.00								
V	− 0.37	0.50	0.86	− 0.12	1.00							
Mo	0.34	− 0.13	− 0.07	− 0.09	− 0.03	1.00						
Li	− 0.43	0.35	0.87	− 0.31	0.92	0.16	1.00					
Co	− 0.12	0.72	0.54	0.36	0.65	− 0.09	0.50	1.00				
Sb	0.24	0.35	− 0.32	0.64	− 0.27	− 0.61	− 0.52	0.30	1.00			
As	− 0.47	0.33	0.56	− 0.42	0.64	− 0.14	0.71	0.29	− 0.23	1.00		
Cd	− 0.19	− 0.05	− 0.33	0.13	− 0.26	− 0.17	− 0.26	0.00	0.07	− 0.23	1.00	
Be	− 0.53	0.37	0.85	− 0.31	0.84	0.06	0.95	0.49	− 0.48	0.63	− 0.16	1.00

Calcium, the major component of the local earth crust, shows a strong correlation with Sr (0.95), Ba (0.74), Cr (0.83) and weak correlation with Mg (0.52) and Ti (0.52). The other elements are not correlated with Ca that suggests that these elements are associated with silicates (Al_2_SiO_5_) rather than the calcium carbonate (CaCO_3_) fraction. On the other hand, Ca is a major element of construction materials (cement, lime, gypsum) and extensively can be found everywhere in the built environment. Since the indoor dust is a mixture of the internally generated particles and those intruded from outside, the actual indoor level of Ca depends on the relative contribution of indoor and outdoor sources that can have a high variation among the locations.

Noted however, no correlation was found for Na, Cu (except with Pb), Mo, Sb and Cd with any elements that indicate that there might be individual (indoor or outdoor) sources for these elements.

### Comparison of indoor dust composition with other local samples

As the correlation analysis of the elements indicated in the previous section, the compositions of the retained indoor dust samples are determined by different—indoor and outdoor—sources. The relative concentrations of the elements can refer to their origin. Metal-to-aluminium ratio (Me/Al) is a widely applied indicator for enrichments or depletions of elements relative to the background composition (Yiǧiterhan and Murray 2008; Yigiterhan et al. 2011) due to the more or less homogeneous geographical distribution of Al in the earth crust. Also, Al is supposed not to be influenced by anthropogenic emissions. Consequently, Al has been ubiquitously used as a reference element in many studies including aerosol composition investigations (Abrahim and Parker 2008; Rushdi et al. 2013, Gomes and Gillette 1993; Shelley et al. 2014).

**Fig. 2:**
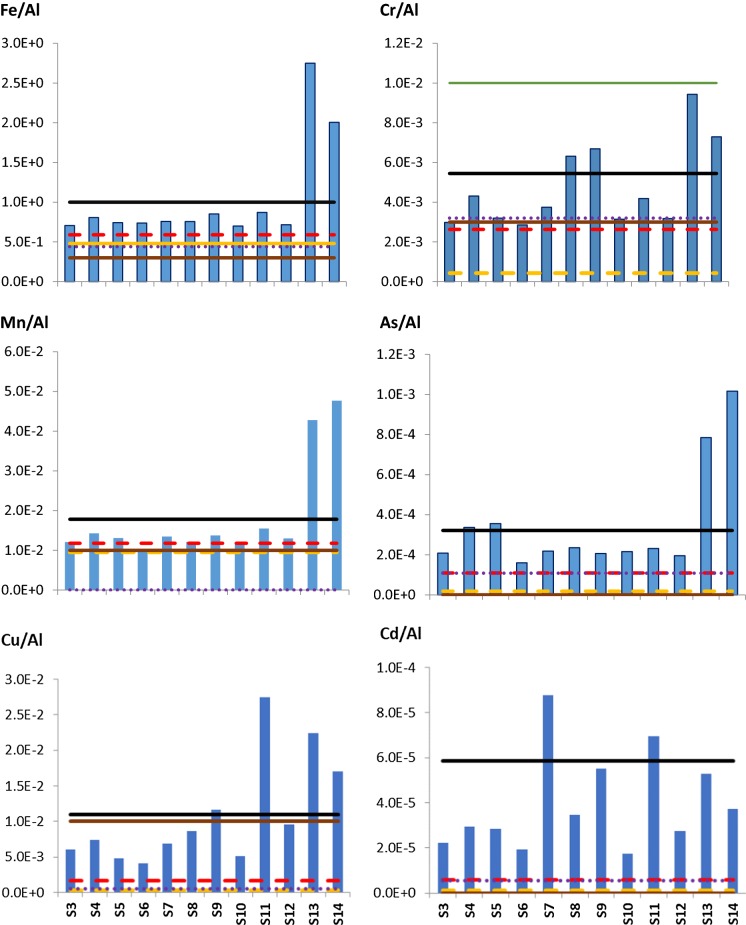

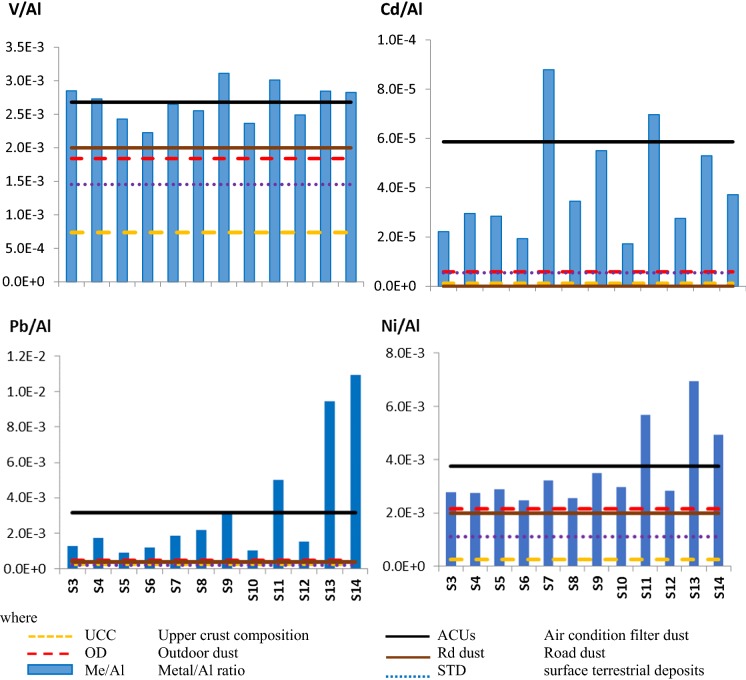
Bar graph for ACUs dust samples showing mean Me/Al ratios in the sample locations. Me/Al elemental ratios, given at *Y*-axis, indicating the indoor enrichments compared to averages of Me/Al ratios of OD, Rd and STD dust (Yigiterhan et al. 2018)

The Me/Al ratios for ten selected elements at the 12 sampling locations are presented in Fig. 2 together with the Me/Al ratios of other local samples such as OD, Rd and STD. Also, the ratios were compared to the UCC as well. Me/Al ratios of these four reference samples were presented as horizontal lines in the figure.

All the presented metals show pronounced enrichment compared to the outdoor samples (OD and Rd) as well as the background compositions (STD, UCC). As it was expected from the correlation analysis Fe/Al, Mn/Al and V/Al ratios show homogeneous distribution among the sampling locations except for the S13 and S14 locations where iron and manganese are more enriched than at other locations. The other metals have significant variation among the sampling locations.

These findings support the idea that indoor dust is primarily transported from outdoor (Bavec et al. 2016; Zheng et al. 2018) and aggravated by indoor sources (Shraim et al. 2016; Tong 1998). The contribution of indoor sources explains the significant spatial variation of the composition even in neighbours’ locations such as S8–S9 or S10–S11.

### Pollution evaluation

For a better understanding of the indoor air pollution composition, enrichment factors (EFs) were calculated using Eq. 1. This environmental index can give a clue for differentiating between anthropogenic and natural sources (Alghamdi et al. 2015; Li et al. 2017). Enrichment factors around 1 indicate the dominance crustal origin, while values higher than ten may indicate anthropogenic contribution (Kamaruzzaman et al. 2009). The higher the EF values, the more severe the anthropogenic influence.

In this study, EFs were used for the characterisation of the degree of elemental enrichments concerning the local surface terrestrial deposits (STD) considered as a background composition. The STD samples were collected from various land surface locations that reflect different proportions of typical Qatari geological formations such as rawdha, sabkha, lithosol, and sandy soils. Soils in Qatar contain small amounts of organic material and are generally calcareous and agriculturally unproductive and have very similar Me/Al ratios as the Qatari aeolian dust for most elements. Qatari STD is composed of two end-members: (1) a calcium carbonate (CaCO_3_) fraction that is relatively depleted in most other elements and (2) an aluminosilicate (Al_2_SiO_5_) fraction with a composition similar to UCC. More details of the STD samples can be found in Yigiterhan et al. (2018).

**Fig. 3:**
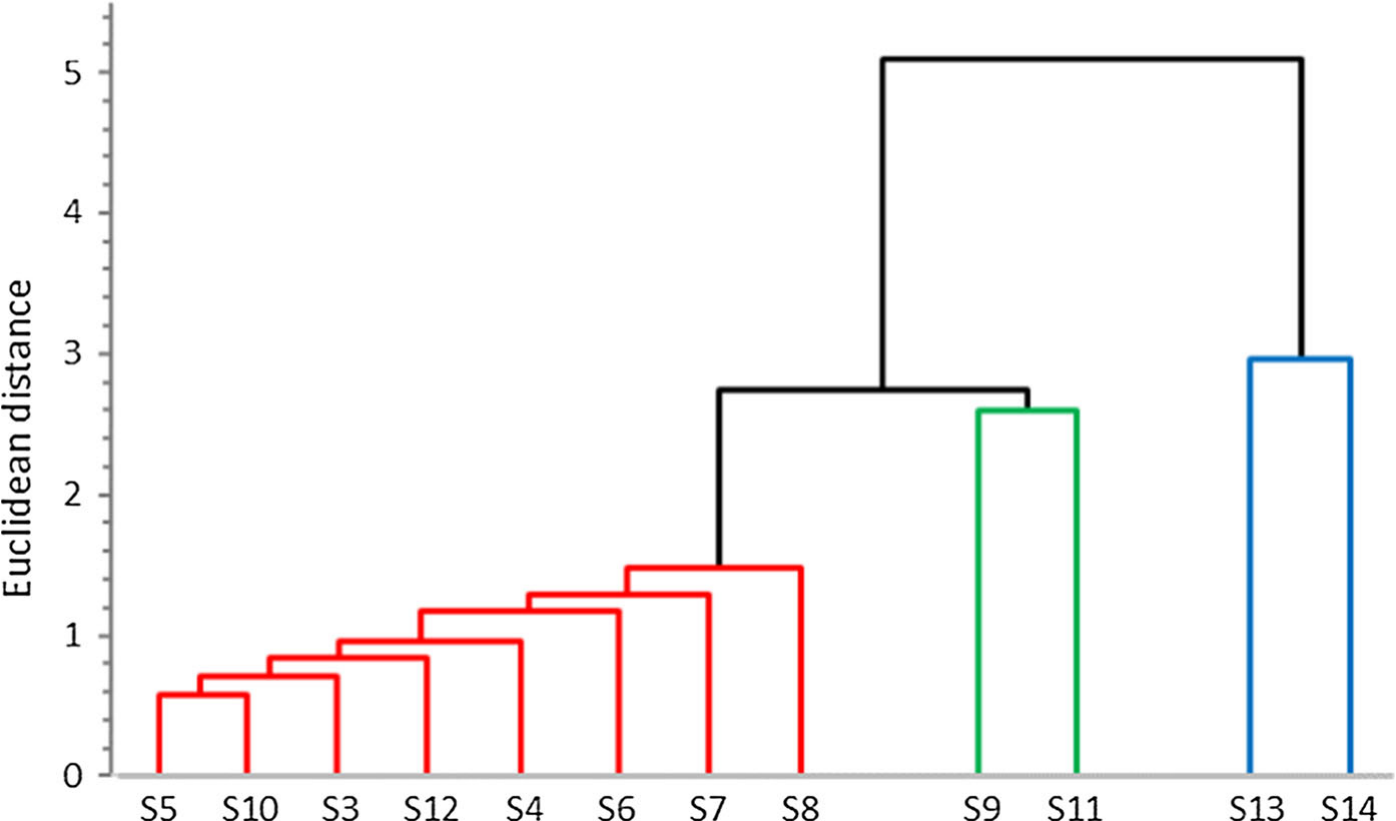
Dendrogram of Me/Al ratios at 12 sampling locations based on Euclidean distance calculation and an average linkage clustering method

The indoor air pollution of the different locations was compared based on similarity analysis using multivariate statistical hierarchical clustering (Idris 2008; Souissi et al. 2001). In this method, each sampling location is represented as a point in the space of 23 dimensions (according to the 23 Me/Al variables). The distances between the points were calculated by the Euclidean distance formula. The two points with the smallest distance were linked, and their average Me/Al ratios were calculated. In the next step, a new distance matrix was calculated within the two linked points which were replaced by their average values (average linkage clustering method). The associated dendrogram that refers to the Euclidean distances between the data points was created using Minitab Software Version 18 (Fig. 3).

In figure, three typical groups can be distinguished. At most of the samples, the Me/Al ratios are close to each other, below the distance of 1.5. This group involves locations of S3, S4, S5, S6, S7, S8, S10 and S12. Locations S9 and S11 form a distinct group with a distance of 2.6. The distance of this group from the previous one is 2.7. The two industrial zone samples form the third group with a distance of 2.8. This group falls far from the previous group averages with a distance of 5.1.

Based on the previous classification of the samples, the EF group averages were calculated. Table 6 summarises the EFs for the three typical indoor environments (from ID1 to ID3) compared to OD and Rd enrichment factors. None of the locations shows enrichments concerning the elements of Ca, Mg, Na, K and Sr, which are the major components of the local earth crust.

**Table 6:** Enrichment factors of the measured elements in ACUs dust for three indoor groups (ID1, ID2, ID3) in comparison to a published outdoor, road dust sample with respect to STD (Yigiterhan et al. 2018)

Element	S3, S4, S5, S6, S7, S8, S10, S12		S9, S11	S13, S14	Outdoor	Road dust
	ID1		ID2	ID3	OD	Rd
Ca	0.94		1.41	1.18	0.97	1.05
Mg	0.97		1.24	1.23	1.11	1.23
Fe	1.69		1.97	5.43	1.35	1.34
Na	0.83		1.32	0.97	1.31	1.12
K	1.34		1.66	1.22	0.87	1.02
Zn	20.7		50.3	131	7.83	16.2
P	3.86		5.57	3.67	2.14	1.92
Sr	0.71		0.97	0.92	1.07	1.02
Mn	1.01		1.17	3.63	0.94	0.98
Ba	1.37		2.14	3.26	0.97	1.45
Cu	12.9		38.4	38.8	3.19	14.3
Cr	1.16		1.70	2.61	0.82	0.9
Ni	2.54		4.15	5.38	1.96	1.63
Pb	7.07		20.0	49.4	2.23	2.12
V	1.75		2.11	1.95	1.27	1.43
Mo	2.53	S12: 76.6	5.93	18.2	3.69	5.05
Li	1.33		1.27	1.69	1.81	0.94
Co	2.05		3.68	6.16	1.19	1.01
As	2.22		2.02	8.32	1.01	–
Cd	4.70	S7: 16.1	11.4	8.24	1.10	–
Be	2.18		1.66	2.01	1.47	1.59

Ti and Sb were not measured in STD samples

The Rd sample shows significant enrichment of Zn (EF = 16.2) and Cu (EF = 14.3), which are two main tracers of the non-combustion traffic emission. Zinc is a component of the rubber tire. Thus, tire debris particles are enriched in Zn, while Cu is emitted to the atmosphere by the ware of brake pads (Apeagyei et al. 2011). The outdoor sample is also enriched by Zn and Cu that indicates the traffic influence on the urban outdoor dust. The EFs are lower compared to the road dust, which is directly affected by the traffic emission. All the indoor samples have significantly higher Zn, Cu and Ni enrichment factors (ranging from 20.7 to 131, 12.9 to 38.8 and 2.54 to 5.38, respectively) than the outdoor sample as a clear sign of the contribution of indoor sources. Rasmussen et al. (2001) studied elemental composition 22 indoor dust and soil samples from one city. They found that Cu, Zn and Ni concentrations were 9, 7 and three times higher in indoor dust than in outdoor soil. Tunno et al. (2016) have reviewed and summarised elemental compositions from different indoor sources. They concluded that the composition of indoor pollution could be attributed to two main source types: (1) combustion sources such as cooking, smoking and candle burning that generating Al-, Cd-, Fe-, Pb-, Mn-, and Zn-rich particles, and (2) other sources such as soil resuspension due to personal activity, hygiene and personal care products, as well as chemical cleaning agents, which are releasing Al-, Cd-, Cu-, Fe-, Pb-, V-, and Zn-rich particles.

Regarding the industrial zone samples (ID3), extremely high Zn enrichment factor was found (EF = 131). Since both offices at the industrial zone were affected by smoking, this high EF can be attributed to cigarette smoke or a workshop emission in the proximity.

Pb and Mo are also enriched in the road dust and outdoor sample due to traffic activity. Regarding the indoor samples, Pb is more enriched than outdoor, especially in the ID2 and ID3 sample groups. In the case of Mo, ID2 group is slightly more enriched than the Rd sample, while ID1 group shows depletion compared to the outdoor samples without considering the S12 sample which has extreme high Mo value (EF = 76.6) that exceeds even the EF from the industrial zone (EF = 18.2). The outlying Mo value of S12 was discharged from the group average and presented separately in the table (i.e. in the split cells of ID1).

Cd shows pronounced enrichment in all indoor samples, especially in ID2 and ID3 sample groups. The Cd EF is the lowest the ID1 group except for S7, where extremely high Cd EF was found (EF = 16.1). The outlying Cd value was discharged from the group average and presented separately in the split cell. The ID3 sample group, which contains samples from the industrial zone, is enriched by Fe, Mn, Ba, Cr, Co, and As. These elements are slightly or not enriched in the other indoor and outdoor samples; thus, we can attribute them to the industrial activity nearby the sampling locations.

## Conclusions

In this study, the elemental composition of indoor dust samples retained by ACUs filters at various locations of Doha city has been analysed. The elemental composition of ACUs dust has been compared to the local outdoor dust deposit, road dust sample and the terrestrial surface composition. The analysed elements cover 20.6% of the total sample mass that is lower than in the case of outdoor samples. Supposing similar mineralogical composition of the particles, the lower mass percentage can be due to higher contribution from organic material originating from indoor sources.

In order to characterise the indoor samples, enrichment factors of the elements were calculated using aluminium as a reference element and STD as a reference composition. Zn, Cu and Ni significantly enriched the indoor ACUs dust samples than the outdoor samples. Also, pronounced enrichment of Pb and Cd was found. The higher enrichment factors compared to the outdoor samples might indicate contributions of indoor emissions.

The collected ACUs filter dust samples show the contribution of outdoor mineral particles, non-exhaust traffic emission, industrial sources, as well as the influence of indoor activity such as smoking.

## Appendix 1: The detailed chemical analysis results of the ACUs filter dust samples

**Table:**

Sample	Al	Ca	Mg	Fe	Na	K	Ti	Zn	P	Sr	Mn	Ba
	%	%	%	%	%	%	%	ppm	ppm	ppm	ppm	ppm
S3	2.39	11.76	3.06	1.69	1.11	0.73	0.18	306	647	466	289	349
S4	2.08	17.23	2.69	1.68	1.19	0.80	0.19	368	458	584	297	313
S5	2.27	10.38	2.27	1.69	1.52	0.93	0.14	257	659	414	297	243
S6	3.23	10.39	2.64	2.39	0.33	0.85	0.23	404	815	349	330	292
S7	1.43	8.11	1.73	1.08	1.22	0.89	0.10	315	589	283	192	184
S8	1.71	14.93	2.27	1.29	1.24	0.76	0.16	636	738	523	208	271
S9	1.52	13.53	2.03	1.30	1.23	0.70	0.37	908	643	427	209	276
S10	1.94	9.16	2.11	1.36	1.06	0.78	0.11	337	465	347	234	207
S11	0.85	6.79	1.42	0.74	0.83	0.45	0.09	457	415	238	132	177
S12	1.98	9.75	2.59	1.42	1.07	0.71	0.14	656	800	390	258	220
S13	1.67	14.30	2.91	4.60	1.34	0.66	0.16	2824	565	528	716	566
S14	2.68	14.87	3.30	5.37	1.35	0.89	0.21	2423	700	585	1275	682
Mean	1.98	11.77	2.42	2.05	1.12	0.76	0.17	824.25	624.50	427.83	369.75	315.00
SD	0.62	3.19	0.55	1.44	0.30	0.13	0.08	865.11	131.28	113.23	320.38	155.19
CV (%)	31.50	27.10	22.80	70.06	27.05	17.05	43.48	104.96	21.02	26.47	86.65	49.27
Range	2.38	10.44	1.88	4.63	1.19	0.48	0.28	2567.00	400.00	347.00	1143.00	505.00

Sample	Cu	Cr	Ni	Pb	V	Mo	Li	Co	Sb	As	Cd	Be
	ppm	ppm	ppm	ppm	ppm	ppm	ppm	ppm	ppm	ppm	ppm	ppm
S3	145	71.4	66.9	30.8	68.2	3.2	14.1	10.0	10.2	4.98	0.53	0.48
S4	153	89.8	57.6	36.5	56.8	4.0	13.7	9.8	12.2	7.01	0.62	0.49
S5	109	72.5	65.9	20.6	55.2	3.2	14.6	9.8	9.8	8.08	0.65	0.62
S6	131	92.2	79.9	38.4	72.0	3.5	18.4	12.8	10.1	5.17	0.63	0.88
S7	98.0	53.6	46.2	26.4	37.9	2.8	9.0	6.6	10.1	3.12	1.26	0.39
S8	149	108	43.6	37.6	43.7	8.7	9.2	7.9	11.7	4.03	0.59	0.39
S9	177	102	53.1	49.1	47.3	6.8	8.9	15.0	14.3	3.14	0.84	0.33
S10	99.1	60.9	57.6	19.6	45.9	4.0	11.1	8.2	11.2	4.19	0.33	0.47
S11	234	35.7	48.4	42.9	25.7	2.6	5.0	4.9	12.2	1.97	0.59	0.14
S12	190	62.9	56.1	30.0	49.3	96.1	13.7	8.4	7.7	3.88	0.55	0.51
S13	374	158	116.4	158.3	47.6	24.7	13.8	25.6	21.5	13.1	0.89	0.33
S14	455	195	132.1	292.9	75.6	22.1	19.7	29.0	23.1	27.2	0.99	0.69
Mean	192.84	91.83	68.65	65.26	52.10	15.14	12.60	12.33	12.84	7.16	0.71	0.48
SD	111.98	45.32	28.01	80.66	14.45	26.60	4.18	7.51	4.72	6.98	0.25	0.19
CV (%)	58.07	49.35	40.80	123.61	27.74	175.68	33.19	60.86	36.74	97.48	35.21	40.08
Range	357.00	159.30	88.50	273.30	49.90	93.50	14.70	24.10	15.40	25.23	0.93	0.74
